# Rate of Osteoporosis Evaluation and Treatment Following Kyphoplasty in Patients With Vertebral Compression Fractures: A Retrospective Study and Review of the Literature

**DOI:** 10.1177/21514593251332463

**Published:** 2025-04-03

**Authors:** Christian Benedict, Avani A. Chopra, Michaela Pitcher, Noel Jeansonne, Edward Fox

**Affiliations:** 1 12310 Penn State College of Medicine, Hershey, PA, USA; 2 Department of Orthopaedics and Rehabilitation, 12311 Milton S. Hershey Medical Center, Penn State College of Medicine, Hershey, PA, USA

**Keywords:** osteoporosis, vertebral compression fracture, kyphoplasty, Fracture Liaison Service, bone mineral density

## Abstract

**Background:**

Lifetime risk of an osteoporotic fracture is 50% for women and 20% for men. Of these fractures, vertebral compression fractures (VCF) are the most common. While surgery plays a crucial role in managing these fractures, preventative measures are also critical when addressing the risk of osteoporotic VCFs. Although many recent guidelines recommend osteoporosis evaluation and treatment for patients with VCFs, the true proportion of patients who undergo an osteoporosis workup following their kyphoplasty procedure is unknown. The aim of this study is to assess the frequency of osteoporosis screening and treatment in patients who undergo a kyphoplasty procedure to correct a vertebral fragility fracture.

**Methods:**

This study utilized the TriNetX Research Network, a database containing de-identified patient information. Using this database, we identified patients from 89 institutions with non-traumatic VCFs and VCFs that resulted from low-energy trauma who subsequently underwent a kyphoplasty procedure. We then analyzed any follow-up osteoporosis treatment or screening they received.

**Results:**

A total of 3371 patients were identified to have undergone kyphoplasty to treat a VCF for the first time. To our knowledge, this is the largest study of its kind to date. Among these patients, 71.3% never had a DEXA scan or prior medical treatment for osteoporosis within 2 years before their kyphoplasty procedure. Additionally, 56.1% of all patients with VCFs treated with kyphoplasty for the first time were never screened or treated for osteoporosis in the two years preceding and 1 year following the procedure.

**Conclusion:**

Our results suggest that only 15.2% of patients with a vertebral fragility fracture secondary to decreased bone density are screened and treated for osteoporosis. Despite existing guidelines recommending osteoporosis evaluation and treatment for patients with VCFs, our findings highlight missed opportunities for intervention. Improving the implementation of existing screening protocols and increasing awareness among healthcare providers could reduce VCF-associated morbidity and mortality.

## Introduction

Osteoporosis is the most common metabolic bone disease in the US and worldwide, with the prevalence in the United States eclipsing more than 50 million patients.^1^ Among Caucasians in the United States over the age of 50, lifetime osteoporotic fracture risk is 50% for women and 20% for men.^2^ These fractures are a significant cause of morbidity and mortality and impose a significant economic strain. It is projected that by 2025, healthcare costs associated with these fractures will contribute upwards of $25 billion in healthcare spending with the incidence expected to continually increase thus further contributing to this cost.^3^

Vertebral compression fractures (VCF) are the most common location for osteoporotic fractures. While these fractures have a large overlap with vertebral fragility fractures, which encompasses fractures resulting from osteoporosis as well as other causes of bone fragility, VCFs are specifically the result of osteoporosis-related weakening of the trabecular bone. While most of these fractures are subclinical, they can cause considerable pain, disability, and deformity impairing mobility and limiting independence, thus reducing quality of life and shortening life expectancy.^4^ Additionally, VCFs are associated with a 5-fold increase in the risk for developing subsequent vertebral fractures.^2^ Patients with severe pain with advanced imaging findings suggestive of a VCF often require surgical vertebral augmentation with vertebroplasty and/or kyphoplasty.^5^ While surgical intervention plays a crucial role in treating these fractures, it is equally important to use preventative measures to help reduce the risk of osteoporotic VCFs from developing. The use of osteoporosis medications such as anti-resorptive agents and bone forming agents have specifically helped to address this by reducing the incidence of spine and hip fractures by up to 50% over 3 years.^6^ Unfortunately, many patients never receive these medications due to lack of screening for osteoporosis and failure to promptly identify osteoporosis and treat it.

Although several guidelines including those from the American Association of Clinical Endocrinology 2020,^7^ The Menopause Society 2021,^8^ and The American College of Radiology 2022^9^ recommend bone density evaluation, particularly if there is concern for VCF, the true proportion of patients who undergo screening or receive osteoporosis treatment following vertebroplasty and/or kyphoplasty remains unknown. The goal of this study was, therefore, to investigate the true frequency of osteoporosis screening and treatment among patients undergoing kyphoplasty for VCFs. Despite current recommendations and the effectiveness of kyphoplasty in treating severe VCFs, we hypothesized that many patients may not receive the appropriate osteoporosis treatment needed to improve long-term outcomes and reduce the risk of subsequent VCFs.

## Methods and Materials

To perform this retrospective analysis, data was collected from the TriNetX Research Network, which contains anonymized electronic medical health records aggregated from 89 healthcare organizations. The use of this database was informed by previous literature.^10,11^ Any datasets generated from TriNetX contain only de-identified patient records and do not include any personal identifiable information. It is compliant with the Health Insurance Portability and Accountability Act and is exempt from Institutional Review Board approval. The TriNetX database allows for querying using International Classification of Disease (ICD-10), Current Procedural Terminology (CPT), Healthcare Common Procedure Coding System (HCPCS), and Normalized Medical Prescription (RxNorm) codes to create patient cohorts.

The database was first queried for all patients who underwent a kyphoplasty procedure involving a single vertebral level (CPT: 1022221) between January 1, 2011, and December 31, 2017. Among these patients, we excluded those with documentation of high-energy trauma, neoplastic pathologic fractures, or periprosthetic fractures occurring within 1 month before or on the same day as their kyphoplasty procedure. A full list of the high-energy trauma excluded from this study including fractures caused by transport accidents, falls from significant heights, and assault-related injuries can be found in within Supplemental 1. For the purposes of this study, any VCF not coded using one of the exclusion criteria listed in Supplemental 1 were included as these cases were considered as low-energy, or occurring in the context of minimal trauma, such as falls from standing height or less, or those associated with underlying conditions including osteoporosis or osteopenia. This definition aligns with clinical presentations commonly seen in elderly patients or those with metabolic bone diseases.

The kyphoplasty cohort was queried to identify patients who had documentation of a dual-energy x-ray absorptiometry (DEXA) scan or had been prescribed osteoporosis medications (supplemental 2) within 2 years and 1 day before the kyphoplasty procedure. This subgroup comprised patients who had received osteoporosis intervention prior to kyphoplasty.

**Figure 1.**
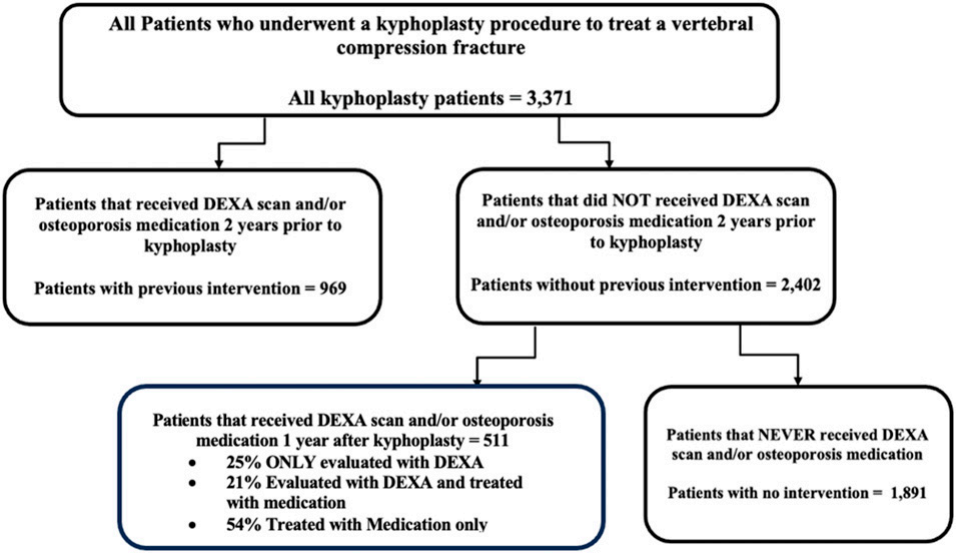
Schematic Demonstrating Cohort Selection.

The patients in the kyphoplasty cohort who were not screened or treated for osteoporosis in the 2 years prior to the procedure were categorized as the treatment-naïve cohort. This cutoff was chosen as bisphosphonates, the osteoporosis medication with the longest acting residual benefit, has been demonstrated to reduce fracture risk for up to two years after being stopped.^12,13^ Furthermore, regarding DEXA screening, it is generally recommended that individuals diagnosed with osteoporosis or at high risk for fractures undergo repeat scans every 1-2 years, as outlined by current guidelines from the American Association of Clinical Endocrinology Physicians.^7^ An additional query was conducted within the treatment-naïve cohort to identify patients who had a DEXA scan or had been prescribed osteoporosis medications within 1 year after their kyphoplasty procedure. The patients that met this criterion formed the follow-up osteoporosis intervention cohort. The treatment-naïve patients who did not receive osteoporosis intervention after their kyphoplasty procedure were classified as the no osteoporosis intervention cohort. (Figure 1) Patient counts and demographics were recorded for each cohort. Additionally, the treatment and treatment naive cohorts were queried a second time to identify any patients who underwent additional kyphoplasty procedures or had subsequent non-vertebral fractures more than 3 months after their initial kyphoplasty.

All statistical analyses were performed using Microsoft Excel version 16.77.1 (2023). Chi-square testing was used for categorical variables, with statistical significance defined as *P* < .05. Confidence intervals were set at 95% for odds ratio calculations assessing non-vertebral fracture incidence across patient cohorts with varying levels of osteoporosis evaluation and/or treatment.

## Results

**Figure 2.**
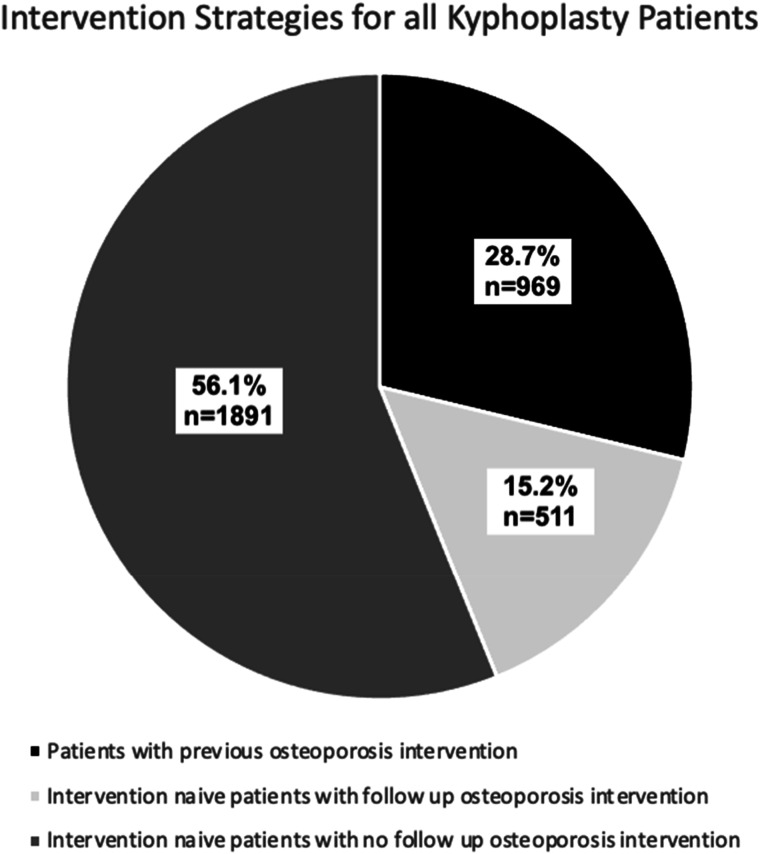
Intervention Strategies for First-Time Kyphoplasty Patients. Intervention was Defined as Either Medical Management or Osteoporosis Screening.

This study identified a total of 3371 patients who underwent kyphoplasty to treat a vertebral compression fracture for the first time. This cohort was comprised of 67.2% females and 31.3% males and had an average age of 78 years. Among these patients, 71.3% were treatment naive and never had a DEXA scan or medical treatment for osteoporosis within two years prior to their kyphoplasty procedure. The average age of this group was 78 years, consisting of 71.9% females and 26.8% males. 56.1% of all patients were never screened or treated for osteoporosis in the 2 years preceding and 1 year following the procedure. This cohort of patients were 63.5% female and 34.9% male and had an average age of 78 years. Only 15.2% of all patients never had a DEXA scan or medical treatment for osteoporosis prior to their procedure but were screened or treated for osteoporosis within 1 year after their surgery. This group of patients were 70.4% female and 27.6% male. Of this cohort, 25.0% were evaluated with DEXA alone, 20.8% were evaluated with DEXA and subsequently prescribed osteoporosis medications, and 54.2% were only prescribed medications. A summary of these data can be found in Table 1, Figures 1 and 2. The medications prescribed following kyphoplasty procedures were bisphosphonates (54%), RANKL inhibitors (21%), calcitonin (12%), PTH analogs (11%), selective estrogen receptor modulators (1%), and sclerostin inhibitors (1%), which is summarized in Table 2.

**Table 1.** Number of Patients and Gender Distribution of Each Cohort.

Cohort	Total Patients (n)	Average age at time of procedure (years)	Female (%)	Male (%)	Unspecified (%)
All kyphoplasty patients	3371	78	67.2	31.3	1.5
Patients with previous osteoporosis intervention	969(28.7%)	77	71.9	26.8	1.3
Intervention naive patients with follow up osteoporosis intervention	511(15.2%)	78	70.4	27.6	2.0
Intervention naive patients with no follow up osteoporosis intervention	1891(56.1%)	78	63.5	34.9	1.6

**Table 2.** Osteoporosis Medications in Patients Treated Following Their First Kyphoplasty.

Medication	Percent of Treatment Naïve Patients Cohort Treated Post-Kyphoplasty
Bisphosphonates	54%
PTH analogs	11%
RANKL inhibitor	21%
Sclerostin inhibitor	1%
Selective estrogen receptor modulator	1%
Calcitonin	12%
Total	100

**Figure 3.**
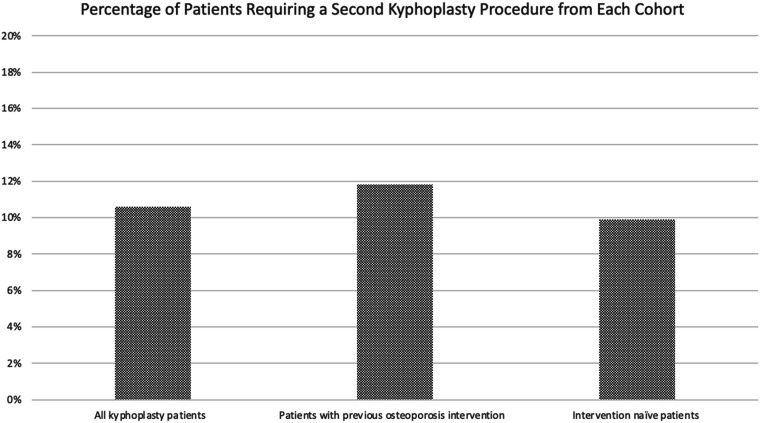
Percentage of Patients From Each Cohort who Underwent a Second Kyphoplasty More than 3 months after Their Initial Procedure. Intervention was Defined as Either Medical Management or Osteoporosis Screening.

Among all patients included in this study, 352 (10.4%) patients had a second kyphoplasty that occurred more than 3 months after their initial procedure. In the cohort of patients with a recent history of osteoporosis treatment, 114 (11.7%) had an additional kyphoplasty procedure. Of all the patients without a recent history of osteoporosis evaluation and/or treatment, 238 (9.9%) had a second kyphoplasty procedure. A chi-square test was performed to compare the two groups, yielding a *P*-value of 0.125, indicating no statistically significant difference. These data can be found in Table 3 and Figure 3.

**Table 3.** Number of Patients With Second Kyphoplasty More Than 3 months After Initial Procedure. Intervention was Defined as Either Medical Management or Osteoporosis Screening. Chi-Square Comparison Between Patients With Previous Osteoporosis Intervention and Intervention-Naïve Patients was Performed, With a *P*-value of 0.125 Indicating No Statistical Significance.

Cohort	Number of Patients with 2nd Kyphoplasty (Percent of Respective Cohort)
All kyphoplasty patients	352 (10.4%)
Patients with previous osteoporosis intervention	114 (11.7%)
Intervention naïve patients	238 (9.9%)

**Figure 4.**
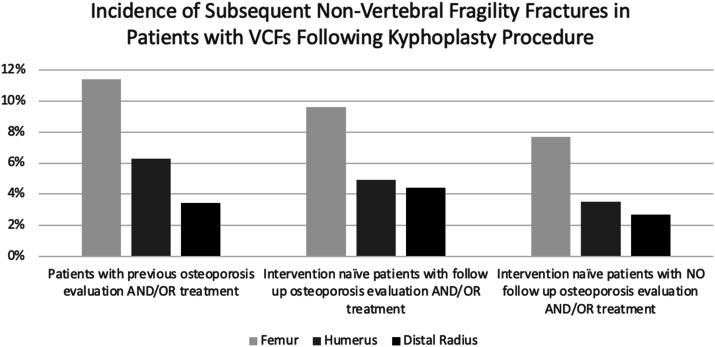
Incidence of Non-vertebral Fragility Fractures in First Time Kyphoplasty Patients.

An analysis of non-vertebral fracture rates in the different cohorts following kyphoplasty revealed several trends. Patients with previous osteoporosis evaluation and/or treatment had the highest fracture rates across all sites, with 11.4% experiencing femur fractures, 6.3% experiencing humerus fractures, and 3.4% experiencing distal radius fractures. In comparison, intervention-naïve patients with follow-up osteoporosis evaluation and/or treatment had slightly lower fracture rates, with 9.6% for femur fractures, 4.9% for humerus fractures, and 4.4% for distal radius fractures. Odds ratios were 0.83 (95% CI: 0.65-1.05) for femur, 0.77 (95% CI: 0.58-1.02) for humerus, and 1.29 (95% CI: 0.94-1.77) for distal radius fractures, suggesting lower risks for femur and humerus fractures, but a higher risk for distal radius fractures when compared to the previously treated cohort. The lowest fracture rates were observed in intervention-naïve patients without follow-up osteoporosis evaluation and/or treatment, with 7.7% for femur fractures, 3.5% for humerus fractures, and 2.7% for distal radius fractures. The corresponding odds ratios were 0.66 (95% CI: 0.51-0.85) for femur, 0.55 (95% CI: 0.40-0.76) for humerus, and 0.79 (95% CI: 0.55-1.14) for distal radius fractures, indicating a reduced risk for fractures in the femur and humerus, but no significant change in risk for distal radius fractures when compared to the previously treated cohort. This data can be found in Table 4 and Figure 4.

**Table 4.** Comparison of Non-Vertebral Fracture Incidence Across Patient Cohorts With Varying Levels of Osteoporosis Evaluation and/or Treatment. Odds Ratios (OR) and 95% Confidence Intervals (CI) Were Calculated Using the Cohort of “Intervention-Naïve Patients With NO Follow-Up Osteoporosis Evaluation AND/OR Treatment” as the Reference Group.

Cohort	Femur (%)	Femur OR (95% CI)	Humerus (%)	Humerus OR (95% CI)	Distal Radius (%)	Distal Radius OR (95%CI)
Patients with previous osteoporosis evaluation AND/OR treatment	11.4%	Reference	6.3%	Reference	3.4%	Reference
Intervention naïve patients with follow up osteoporosis evaluation AND/OR treatment	9.6%	0.83 (0.65-1.05)	4.9%	0.77 (0.58-1.02)	4.4%	1.29 (0.94-1.77)
Intervention naïve patients with NO follow up osteoporosis evaluation AND/OR treatment	7.7%	0.66 (0.51-0.85)	3.5%	0.55 (0.40-0.76)	2.7%	0.79 (0.55-1.14)

## Discussion

Vertebral compression fractures, especially those precipitated by osteoporosis, are a widespread issue, particularly within the elderly population. Consequently, our primary objective was to evaluate the prevalence of osteoporosis screening and treatment in patients undergoing a kyphoplasty procedure in the management of their VCF. To our knowledge, this study is the largest to date examining these rates. Our results suggest that only a small proportion of patients with a vertebral compression fracture secondary to decreased bone density are screened and treated for osteoporosis. In fact, among patients never treated or screened for osteoporosis, only 21% received any osteoporosis screening or treatment following their first kyphoplasty procedure.

The inadequacy of follow-up treatment for osteoporosis has been well documented in the setting of VCFs. In a 2021 study, Haffner et al found that in a population of patients with low-energy VCFs, only 11.5% received post-injury DEXA evaluation and only 19.2% received appropriate osteoporosis-targeting pharmacological therapy.^14^ Similarly, a study of 156 former military members with VCFs revealed only 38% were treated medically for osteoporosis in the year following their fracture and 39% patients underwent DEXA evaluation.^15^ In another large-scale study of 2933 VCF patients performed by Barton et. Al, 98% of patients did not undergo a DEXA scan within the two years before or 1 year following their VCF. Additionally, only 7% were prescribed antiresorptive therapy following their fracture, with 73% of patients never receiving any antiresorptive therapy at all. This study also revealed that 38% of patients went on to develop a second fragility fracture within 2 years. The authors did acknowledge, however, that their figures may be confounded as they did not differentiate between high impact and low impact VCFs in their study population.^16^ The profoundly low treatment and screening follow-up rates for VCFs occur despite these fractures being associated with a 5 times greater risk of developing future VCFs and significantly increased mortality rates.^2,17,18^

Although the problem with inadequate follow-up is likely a multifactorial issue, previous literature has attributed this in part to the fact that there is rarely a specific specialist to take responsibility for follow-up care, thus leading to inadequate post-procedural treatment.^19^ The treatment is often shared between emergency medicine physicians, spine surgeons, primary care physicians, and endocrinologists, which introduces potential communication gaps for follow-up details to be overlooked. Although many specialists may be involved in VCF treatment, Daffner et al found that orthopaedic spine surgeons tend to address osteoporosis or refer patients for osteoporosis follow-up at rates that are higher than other physicians who treat VCFs including neurosurgeons, interventional radiologists, and pain management physicians. The study cited a few potential reasons for this discrepancy including the fact that most osteoporosis advocacy programs are primarily aimed at orthopaedic surgeons. Furthermore, they rationalized that because orthopaedic surgeons deal with other fragility fractures such as those of the extremities, they may be better primed to treat osteoporosis and are thus more likely to refer their patients for follow-up.^20^ These findings reveal the importance of involving all specialists who treat VCFs in osteoporosis advocacy programs.

It is also possible that lack of follow up in osteoporosis treatment may reflect lack of resources and facilities available to patients for screening. Some institutions have addressed these poor follow-up rates by establishing a Fracture Liaison Services. These programs help to identify at-risk individuals and implement tailored treatment plans to successfully reduce major bone re-fractures by 40%, any bone re-fractures by 30%, and mortality rate two years after injury by 35% when compared to those not evaluated by the FLS, thus representing a potential solution to the poor follow up-rates associated with VCFs.^20,21^ Similarly, Gonzalez-quevedo et al found in addition to decreased rates of refracture, the implementation of a FLS markedly reduced care costs.^22^ Barton et al also demonstrated that the implementation of a FLS resulted, on average, in a six-fold improvement in DEXA scanning rates and a three-fold improvement in antiresorptive therapy rates.^23^ Other initiatives, such as the American Orthopaedic Association’s *Own the Bone* project, have worked to improve patient counseling on calcium and vitamin D supplementation, exercise, fall prevention, and communication between specialists and PCPs. These factors have all contributed to reduced rates of refracture and improved patient follow-up and should be considered when dealing with patients who have VCFs.^24^

Despite constituting 31.3% of all patients who underwent kyphoplasty for vertebral compression fractures, males comprised 34.9% of the patients who did not receive any pre- or post-kyphoplasty osteoporosis treatment. In contrast, only 26.8% of patients who received pre-kyphoplasty osteoporosis treatment were males. This discrepancy may partly be attributable to the United States Preventive Services Task Force (USPSTF) not recommending routine osteoporosis screening for men due to insufficient evidence regarding its benefit in preventing osteoporotic fractures.^25^ Osteoporosis is four times more common in women, corresponding to a greater focus on female populations and resulting in a lack of extensive research on male osteoporosis screening.^26^ However, it is crucial to recognize that men are also at substantial risk for fragility fractures. In fact, current evidence indicates that men experience more osteoporosis-related complications and exhibit a higher mortality rate following osteoporosis fractures, particularly those of the hip, than women, starting from the time of hospital admission.^27-30^ This emphasizes the importance of developing targeted screening protocols for men to address this critical gap in osteoporosis management.

Our study also evaluated refracture rates by examining the proportion of patients who underwent additional kyphoplasty procedures or had other, non-vertebral fractures more than 3 months after their initial kyphoplasty. We hypothesized that the refracture rate would be higher in the treatment-naive group compared to the cohort of patients with a history of osteoporosis intervention. Contrary to our expectations, we found that the treatment-naive group had a statistically insignificant (*P* = .125), proportion of patients undergoing a second kyphoplasty, indicating a similar refracture rate to the group that underwent treatment and/or screening for osteoporosis. Furthermore, the data in Table 4 and Figure 4 revealed that patients without follow-up osteoporosis evaluation or treatment had significantly lower rates of femur and humerus fractures compared to those with prior osteoporosis evaluation and/or treatment. While this may be a function of underdiagnosis or misclassification in this group leading to fewer documented fractures, it is also possible that patients who did not receive follow-up osteoporosis treatment after kyphoplasty may represent a healthier subset with lower baseline fracture risk. The unexpected refracture rates may also be related to the small treatment group used in our analysis therefore making the rate of refracture difficult to truly measure. Furthermore, the use of a database with deidentified patient information limited our ability to fully elucidate the cause behind this finding. Existing literature generally indicates that refracture rates are higher in treatment-naive patients, highlighting the need for further research to understand these discrepancies.^31,32^

One of the biggest strengths of this study was its size. To our knowledge, it is the largest of its kind to date. Another strength of our study is the accessibility of ICD-10 and CPT codes post-treatment which allowed for the collection of discrete, identifiable data points and enabled the monitoring of intervention rates. There are also several limitations of this study. One limitation is that this is a retrospective study that utilizes de-identified EMR data. The data in the TriNetX platform reflects how information is entered into the patients’ EMR during care; therefore, there is no guarantee of completeness. For example, average patient BMI and vertebral level treated could not be obtained as less than half of our patient cohort had this documented appropriately. Another limitation of this study was that we defined treatment naïve patients as those that had not received treatment for at least two years prior to their kyphoplasty procedure. It is possible, however, that a small subset of patients may have been treated with bisphosphonates greater than two years prior to the procedure and were still benefiting from the therapeutic effects.^12,13^ Similarly, the differing efficacies of osteoporosis medications prescribed post-kyphoplasty represent another limitation. While this study did not analyze the specific effects of individual medications, we have provided epidemiological data on medication use within our cohort for added context (Table 2).

This study demonstrated that there is a significant lapse in osteoporosis intervention following a kyphoplasty for a VCF. Future research should seek to compare refracture rates among patients who underwent osteoporosis screening and/or treatment following kyphoplasty to those that received no intervention. Furthermore, it is crucial to identify the extent to which screening for and/or treating osteoporosis following a kyphoplasty procedure reduces the risk of developing future VCFs.

## Conclusion

Osteoporotic fractures are a significant cause of morbidity, mortality, and economic burden. Of these fractures, vertebral compression fractures (VCF) are the most common. Our results indicate a significant gap in osteoporosis care following kyphoplasty procedures in the management of VCFs. This highlights the importance of adjusting treatment algorithms for patients following kyphoplasty procedures to enable greater prevention of future fractures and morbidity. It also sheds light on the necessity of identifying patients at risk for VCFs including the use of third-party resources such as Fracture Liaison Services to help reduce rates of refracture in patients who undergo kyphoplasty.

## Supplemental Material

Supplemental Material - Rate of Osteoporosis Evaluation and Treatment Following Kyphoplasty in Patients With Vertebral Compression Fractures: A Retrospective Study and Review of the Literature Supplemental Material for Rate of Osteoporosis Evaluation and Treatment Following Kyphoplasty in Patients With Vertebral Compression Fractures: A Retrospective Study and Review of the Literature by Christian Benedict, Avani A. Chopra, BS, Michaela Pitcher, Noel Jeansonne, MD, and Edward Fox in Geriatric Orthopaedic Surgery & Rehabilitation

## Data Availability

The datasets generated during and/or analyzed during the current study are available from the corresponding author on reasonable request.
