# Corrigendum to “Rate of Osteoporosis Evaluation and Treatment Following Kyphoplasty in Patients With Vertebral Compression Fractures: A Retrospective Study and Review of the Literature”

**DOI:** 10.1177/21514593261453503

**Published:** 2026-07-15

Benedict C, Chopra AA, Pitcher M, Jeansonne N, Fox E. Rate of Osteoporosis Evaluation and Treatment Following Kyphoplasty in Patients With Vertebral Compression Fractures: A Retrospective Study and Review of the Literature. *Geriatr Orthop Surg Rehabil*. 2025;16. doi:10.1177/21514593251332463

A reader contacted the Journal with concerns regarding a discrepancy between the noted results in the abstract of the article versus the results section of your article. Specifically, the conclusion section of the abstract, states that “only 15.2% of patients ... are screened and treated for osteoporosis.” However, the results section and Figure 2 indicate that this 15.2% represents patients who were screened or treated for osteoporosis.

Sage and the Journal Editors conducted an internal review of the article and contacted the authors for comments.

The authors have clarified that the 15.2% figure represents patients who were screened or treated for osteoporosis, consistent with the results section and Figure 2. I agree that the wording in the abstract conclusion is imprecise and should be revised to reflect this accurately.

The Conclusion section of the abstract has been update to read.

Our results suggest that only 15.2% of patients with a vertebral fragility fracture secondary to decreased bone density are screened or treated for osteoporosis. Despite existing guidelines recommending osteoporosis evaluation and treatment for patients with VCFs, our findings highlight missed opportunities for intervention. Improving the implementation of existing screening protocols and increasing awareness among healthcare providers could reduce VCF-associated morbidity and mortality.

The Journal Editor approved the changes to the article.

