# Supplementary material for: Rate of Osteoporosis Evaluation and Treatment Following Kyphoplasty in Patients With Vertebral Compression Fractures: A Retrospective Study and Review of the Literature
Source: Geriatr Orthop Surg Rehabil. 2025 Apr 3;16:21514593251332463. doi: 10.1177/21514593251332463 (PMC11970096; doi:10.1177/21514593251332463)
Supplement: Supplemental Material - Rate of Osteoporosis Evaluation and Treatment Following Kyphoplasty in Patients With Vertebral Compression Fractures: A Retrospective Study and Review of the Literature [file sj-pdf-1-gos-10.1177_21514593251332463.pdf]

Supplemental 1: ICD-10 codes to exclude trauma, pathologic fracture, or periprosthetic fracture

C79.5 Secondary malignant neoplasm of bone and bone marrow
M84.4 Pathological fracture, not elsewhere classified
M84.5 Pathological fracture in neoplastic disease
M97 Periprosthetic fracture around internal prosthetic joint
V98-V99 Other and unspecified transport accidents
V95-V97 Air and space transport accidents
V90-V94 Water transport accidents
V80-V89 Other land transport accidents
V70-V79 Bus occupant injured in transport accident
V60-V69 Occupant of heavy transport vehicle injured in transport accident
V50-V59 Occupant of pick-up truck or van injured in transport accident
V40-V49 Car occupant injured in transport accident
V30-V39 Occupant of three-wheeled motor vehicle injured in transport accident
V20-V29 Motorcycle rider injured in transport accident
V10-V19 Pedal cycle rider injured in transport accident
V00-V09 Pedestrian injured in transport accident
W17 Other fall from one level to another
W16 Fall, jump or diving into water
W15 Fall from cliff
W14 Fall from tree
W13 Fall from, out of or through building or structure
W12 Fall on and from scaffolding
W11 Fall on and from ladder
W10 Fall on and from stairs and steps
W09 Fall on and from playground equipment
Y09 Assault by unspecified means
Y08 Assault by other specified means
Y07 Perpetrator of assault, maltreatment and neglect
Y04 Assault by bodily force
Y03 Assault by crashing of motor vehicle
Y02 Assault by pushing or placing victim in front of moving object
Y01 Assault by pushing from high place
Y00 Assault by blunt object
Z04.1 Encounter for examination and observation following transport accident

Supplemental 2: Codes for Osteoporosis Medications

A. RxNorm

36118 salmon calcitonin
1311287 calcitonin
32915 teriparatide
1921069 abaloparatide
2123126 romosozumab
993449 denosumab
73056 risedronate
77655 zoledronic acid
72143 raloxifene
46041 alendronate
42682 etidronate
115264 ibandronate
11473 pamidronate

B. HCPCS

J0630 Injection, calcitonin salmon, up to 400 units

C. CPT

82308 Calcitonin
